# Passive smoking and the risk of hypertension in nonsmoking adults: a systematic review and meta-analysis

**DOI:** 10.7717/peerj.20639

**Published:** 2026-01-14

**Authors:** Yang Song, Ke Du, Huanling Jiang

**Affiliations:** 1 Yantai Nurses School of Shandong, Yantai, China; 2 Department of Emergency, Qingdao Hospital, University of Health and Rehabilitation Sciences (Qingdao Municipal Hospital), Qingdao, China

**Keywords:** Passive smoking, Hypertension, Non-smoking adults, Meta analysis

## Abstract

**Background:**

Previous studies on the association between passive smoking and hypertension are controversial. The association between these two elements remains inconclusive and requires a comprehensive meta-analysis. We conducted a systematic review and meta-analysis to explore this association.

**Methods:**

We searched for full articles from four databases, including PubMed, Web of Science, Embase, and Cochrane databases, from 1971 until February 2025. Cross-sectional, case-control, and cohort studies examining the relationship between passive smoking exposure and the occurrence of hypertension were considered to be suitable for general analysis. Effect sizes and relevant 95% confidence intervals (CIs) were pooled and calculated.

**Results:**

We included 13 studies, with 783,798 nonsmoking adults being included in the pooled analysis An association between passive smoking and elevated risk of hypertension was observed in cross-sectional/case-control studies (Effect size = 1.20, 95% CI [1.08–1.34], *p* = 0.001, *I*^2^ = 99.1%) and in cohort studies (Effect size = 1.17, 95% CI [1.11–1.25], *p* < 0.001, *I*^2^ = 0%). The result was still significant for cross-sectional/case-control studies after excluding two studies based on sensitivity analysis (Effect size =1.29, 95% CI [1.08–1.54], *p* = 0.005, *I^2^* = 73.5%). Subgroup analysis indicated that the increased risk was effective for both male and female populations. For the frequency and duration of secondhand smoking (SHS) exposure, only exposure ≥3 times/week (Effect size = 1.13, 95% CI [1.03–1.24], *p* = 0.012, *I*^2^ = 64.8%) and ≥10 years (Effect size = 1.21, 95% CI [1.13–1.29], *p* < 0.001, *I*^2^ = 0.0%) contributed to an increased risk of hypertension. Subgroup of hypertensive individuals defined by physical examination or self-reported diagnosis showed increased risk (Effect size = 1.15, 95% CI [1.09–1.22], *p* < 0.001, *I*^2^ = 28.8%), but not for those defined by a structured questionnaire.

**Conclusion:**

Exposure to passive smoking is significantly associated with an increased risk of hypertension in both cross-sectional/case-control and cohort studies, and for both male and female populations. Exposure ≥3 times/week and ≥10 years may have an adverse influence on hypertension.

## Introduction

Hypertension is regarded as a significant public health concern, which affects approximately 30% adults worldwide (NCD Risk Factor Collaboration (NCD-RisC), 2021; Mills, Stefanescu & He, 2020). It is considered the leading cause and major risk factor for coronary artery disease, heart failure, stroke, and peripheral artery disease (van den Hoogen et al., 2000; MacMahon et al., 1990; Turana et al., 2021*)*. In addition, it significantly contributes to sudden cardiac death (Shenasa & Shenasa, 2017; Pan et al., 2020). However, most hypertensive individuals are unaware of the disease, which results in insufficient treatment. A previous study conducted in China reported that the rates of prevalence, awareness, treatment, and control of hypertension were 27.8%, 31.9%, 26.4%, and 9.7%, respectively, between 2013 and 2014 (Li et al., 2017). Even for those individuals who have received appropriate diagnoses and treatments, many patients do not adhere to persistent and standard treatment. The global prevalence of inadequate antihypertensive medication compliance ranges from 27% to 40%, which is more common in low- and middle-income countries, as well as non-Western countries (Lee et al., 2022). Given the lack of awareness and severe consequences, it is vital to identify important risk factors and prevent the onset of hypertension.

Extensive research has focused on the relationship between smoking and blood pressure (BP), the main aspects of which include exploring the causal relationship between smoking habits and the incidence of hypertension, as well as examining the influence of smoking on the prognosis of hypertensive patients. Significant increases in BP and heart rate (HR) have been observed following acute cigarette smoking (Cryer et al., 1976; Grassi et al., 1994). With respect to the influence of chronic smoking, existing data are unable to clearly demonstrate a direct causal link between these two conditions. However, several studies have demonstrated significant results. For example, a positive correlation between the duration of smoking and systolic blood pressure (SBP) was demonstrated (Herath et al., 2022), also in aged male individuals (Primatesta et al., 2001). A reduction in or cessation of smoking by switching to e-cigarettes may yield long-term SBP reductions, particularly in hypertensive smokers (Farsalinos et al., 2016). Cigarette smoking elevated daytime and average 24-h BP and HR, likely due to reduced parasympathetic nerve activity (Ohta et al., 2016). Notably, 1 week of smoking cessation significantly lowered 24-h BP compared to continued smoking (Minami, Ishimitsu & Matsuoka, 1999). Smokers also exhibited higher daytime ambulatory BP than nonsmokers, though this difference was absent at night (Verdecchia et al., 1995).

Passive smoking, which is also referred to as environmental tobacco smoking (ETS) or secondhand smoking (SHS), continues to emit multiple harmful substances. Globally, the estimated prevalence of SHS is approximately 40% among children, 33% among male nonsmokers, and 35% among female nonsmokers (Oberg et al., 2011). Throughout the world, SHS is estimated to account for 1% of total mortality, with ischemic heart disease contributing to approximately two-thirds of this attributable burden (Oberg et al., 2011). Under specific conditions, SHS exposure induces nearly equivalent physiological effects to those of chronic active smoking (Barnoya & Glantz, 2006; Argacha et al., 2008). Likewise, the protection of nonsmokers from exposure to SHS leads to a significant decline in cardiovascular mortality (Ong & Glantz, 2004). Therefore, SHS may be a key risk factor for cardiovascular diseases, including hypertension. Previous meta-analyses have reported that neither active nor passive cigarette smoking is related to the emergence of hypertension in children and adolescents (Aryanpur et al., 2019). However, passive smoking is related to a higher SBP (Aryanpur et al., 2019). For nonsmoking adults, research on the impact of passive smoking on hypertension has yielded conflicting findings. A number of studies have documented a severely increased risk of hypertension in nonsmoking adults (Ciruzzi et al., 1998; Leone, 2011; Yang et al., 2017; Tamura et al., 2018; Kim et al., 2019; Akpa et al., 2021; Cao et al., 2024). However, several studies have demonstrated neutral results (Wang et al., 2021; Shen et al., 2021; Jalali et al., 2022). Moreover, other studies have reported different results stratified by sex (Park et al., 2018) and frequency of exposure (Li et al., 2015). Given the discrepancies observed among the published studies, we performed a systematic review and meta-analysis to investigate the pooled effect between passive smoking and hypertension among nonsmoking adults.

## Materials and Methods

### Study selection

This study was registered on PROSPERO (ID: CRD42025640660, https://www.crd.york.ac.uk/PROSPERO/view/CRD42025640660). We conducted a comprehensive literature search among various databases, including PubMed, Embase, Web of Science, and the Cochrane Library, with the search being conducted from 1971 to February 2025. The search aimed to identify peer-reviewed articles published in English that had explored the potential link between passive smoking and the development of hypertension in nonsmoking adults. The following terms were used in the search strategy: (“tobacco smoke pollution” OR (“tobacco” AND “smoke” AND “pollution”) OR “tobacco smoke pollution” OR (“passive” AND “smoking”) OR “passive smoking”) AND (“hypertense” OR “hypertension” OR “hypertension” OR “hypertensions” OR “hypertensions” OR “hypertensive” OR “hypertensives” OR “hypertensives”) AND (“adult” OR “adult” OR “adults” OR “adults”). The review protocol has neither been made public nor uploaded online.

### Criteria for inclusion and exclusion

The following inclusion criteria were utilized and presented in the Population, Exposure, Comparator, and Outcome (PECO) format: (a) the study population under consideration was composed of nonsmoking adults aged over 18 years; (b) the exposure was defined as passive smoking, SHS or ETS; (c) the comparison was performed between individuals who were subjected to passive smoking, SHS or ETS and those who were not; and (d) the outcome of interest was the onset of hypertension, which was defined as self-reported diagnosis, physical examination [SBP ≥ 140 mmHg and/or diastolic blood pressure (DBP) ≥ 90 mmHg] or the current administration of antihypertensive medication. The following exclusion criteria were utilized: (a) items such as case reports and case series, as well as correspondences in the form of letters, review articles, practice guidelines, research protocols, replies to previously published works, and abstracts from conferences; (b) the population of interest was not being currently exposed to passive smoking; (c) studies that failed to accurately report original data or demonstrating data that could not be calculated to obtain the outcome; (d) non-English articles; and (e) fundamental science studies or experimental research.

### Study selection, data collection, and data extraction

The process of data extraction was separately performed by two researchers (Yang Song and Ke Du) during February 2025. Both reviewers (Yang Song and Ke Du) conducted a comprehensive review of all of the possible studies. Huanling Jiang extracted and summarized the relevant information in one table, which included the main characteristics of all of the studies (Table 1). The abovementioned basic information was then double-checked and cross-verified by Yang Song. The senior author (Huanling Jiang) resolved the discrepancies during the entire review process. The Newcastle-Ottawa Scale (NOS) (Wells, O’Connell & Peterson, 2000) served as the tool for assessing study quality (Table S1). Studies that received scores ranging from 7 to 9 were classified as being of high quality. Those studies with scores within the range of 4 to 6 were categorized as having moderate quality. Furthermore, studies scoring between 0 and 3 were evaluated as being of low quality.

**Table 1:** Main characteristics of the included studies.

Study	Study design	Origin	Exposure	Definition of hypertension	Characteristics of participants	OR or RR (95%CI)	Adjusted confounding factors	NOS score
Ciruzzi et al. (1998)	Case-control study	America	Passive smoking from relatives	Structured questionnaire	336 never-smokers with AMI and 446 never-smokers admitted to the same network of hospitals	OR: 3.28 [2.02–5.34]	Age, gender, cholesterolemia, diabetes, hypertension, BMI, years of education, socioeconomic status, exercise and family history of myocardial infarction	6
Li et al. (2015)	Cross-sectional study	Asia	SHS on at least one occasion per week, for at least 30 min per occasion, at home or in public places	Physical examination or self-reported diagnosis	405 non-smoking women were enrolled (age range: 33–82 years)	OR: 1.99 [1.16–3.39]	Age, BMI, education, occupation, menopause, drinking status, and physical activity	6
Wu et al. (2017)	Cross-sectional study	Asia	Environmental smoke on at least one occasion per week, for at least 30 min per occasion	Physical examination or self-reported diagnosis	Local 1078 non-smoking female residents aged 60 years or above	OR: 1.38 [1.03–1.85]	Age, educational level, marital status, physical activity, drinking status, BMI, family history of CVD, treatment of diabetes and hyperlipidemia	6
Yang et al. (2017)	Cross-sectional study	Asia	Exposure to environmental tobacco smoke from husband	Physical examination or self-reported diagnosis	5,027,31 non-smoking females aged 20 to 49 years old	OR: 1.28 [1.27–1.30]	Age, urban inhabitants, higher school education, BMI, and alcohol consumption	6
Tamura et al. (2018)	Cross-sectional study	Asia	SHS in the home or in office	Physical examination or self-reported diagnosis	32,098 lifetime non-smokers (7,216 men and 24,882 women)	OR: 1.11 [1.03–1.20]	Age, sex, alcohol consumption, education level, BMI, physical activity, psychological stress, sleeping hours, family history of hypertension in father and mother, diabetes mellitus, dyslipidemia, menstruation status, and study area	7
Park et al. (2018)	Cross-sectional study	Asia	Passive smoking in the work place and home	Physical examination or self-reported diagnosis	10,532 (women 8987 and men 1,545) never-smokers aged above 20 years old	OR: 1.50 [1.10-2.04) (Women)	Age, height, weight, waist circumference, serum triglyceride, fasting glucose, education, occupation, alcohol intake and marital status	7
						OR: 0.93 [0.52–1.68] (Men)		
Kim et al. (2019)	Cohort study	Asia	Passive smoking indoors at home or the workplace	Physical examination or self-reported diagnosis	108,354 self-reported never-smokers	RR: 1.16 [1.08–1.24]	Age, waist circumference, BMI, frequency of alcohol drinking, frequency of vigorous exercise, glucose, creatinine, uric acid, total cholesterol, HDL, LDL, triglyceride, and high-sensitivity CRP	7
Akpa et al. (2021)	Cross-sectional study	America	Passive smoking from any form of cigarette in any indoor area	Physical examination or self-reported diagnosis	3,067 non-smoking adults (1961 women and 1106 men)	OR: 1.038 [1.037–1.040]	Sex, age, race, employment, income, marital status, alcohol use, and BMI	7
Wang et al. (2021)	Cross-sectional study	Asia	SHS during childhood	Structured questionnaire	2,120 Chinese non-smoking women	OR: 0.96 [0.76–1.20]	Age, obesity, education status, physical activity, alcohol consumption and current SHS exposure status	6
Kim, Kang & Kim (2021)	Cohort study	Asia	Passive smoking indoors at home and in the workplace	Physical examination or self-reported diagnosis	87,486 self-reported and cotinine-verified never smokers without hypertension were included with a median follow-up of 36 months	HR: 1.29 [1.06–1.57] (New SHS)	Age, sex, BMI, waist circumference, vigorous exercise, alcohol consumption, and presence of diabetes creatinine, uric acid, total cholesterol, HDL, LDL, triglycerides, and high-sensitivity CRP	8
						HR: 1.18 [1.01–1.38] (Sustained SHS)		
Shen et al. (2021)	Cross-sectional study	Asia	SHS in the last 7days	Physical examination or self-reported diagnosis	2,826 nonsmoking and nonpregnant women 20-44 years old	OR: 1.12 [0.88–1.44]	Age, race, education level, marital status, BMI, alcohol use, physical activity, diabetes, kidney disease	6
Jalali et al. (2022)	Cross-sectional study	Asia	Husband opiate smoking	Physical examination or self-reported diagnosis	1,541 women (35–70 years) whose husbands smoke opiate regularly started after marriage	OR: 1.05 [0.73–1.51]	Age, education years, BMI, MET and family history of hypertension	6
Cao et al. (2024)	Cross-sectional study	Asia	SHS for more than one day per week at home, at work or in public places	Physical examination or self-reported diagnosis	30,778 never-smokers aged 35–74 years	OR: 1.09 [1.03–1.16]	Age and sex, nationality, BMI, degree of education, marital status, occupation, household income, drinking status, and physical activity	7

**Note:**

Abbreviations: OR, odds ratio; RR, relative risk; HR, hazard ratio; CI, confidence interval; NOS, Newcastle-Ottawa Scale; SHS, secondhand smoke; AMI, acute myocardial infarction; BMI, body mass index; CVD, cardiovascular disease; HDL, high density lipoprotein cholesterol; LDL, low density lipoprotein cholesterol; CRP, C-reactive protein; MET, metabolic equivalent of task.

### GRADE assesement

We used the GRADE system to verify the certainty of the evidence *via* online GRADEpro software (https://www.gradepro.org/) (Atkins et al., 2004), which was also described in other studies (Wang, Wang & Wang, 2025). We considered the following aspects: the limitations inherent in the study design, the potential risk of bias, the degree of inconsistency observed among the different studies, the level of indirectness of the evidence, the issue of imprecision, and any other pertinent factors (Guyatt et al., 2008). Therefore, five degrees of evidence were generated, ranging from high quality to no evidence.

### Statistical analysis

All of the analyses were performed using STATA 17.0 (StataCorp LLC, College Station, TX, USA). Odds ratios (ORs), hazard ratios (HRs), risk ratios (RRs), and other computable data, in conjunction with the respective 95% confidence intervals (CIs), were pooled using the DerSimonian-Laird random effects models. We conducted subgroup analyses according to sex, frequency and duration of SHS exposure, as well as definition of hypertension. We utilized the *I*^*2*^ statistic to assess heterogeneity. A funnel plot was used to verify the existence of publication bias. To identify the potential sources of heterogeneity, sensitivity analysis was performed.

## Results

### Characteristics of entered studies

**Figure 1:**
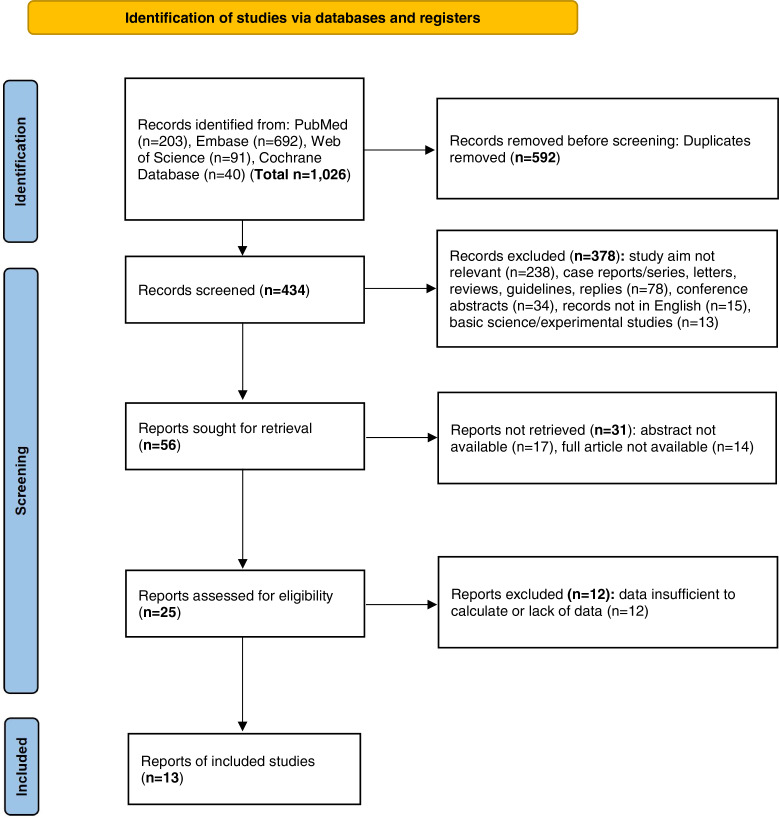
PRISMA flow diagram of the general selection process.

We initially conducted a search and retrieved a total of 1,026 articles, of which 592 duplicate articles were removed from this pool. Upon screening of the records, 378 items were excluded based on various justifications. Specifically, 238 items were excluded because their study objectives were not relevant. Additionally, 78 nonoriginal items were removed. Moreover, 34 conference abstracts, 15 articles that were not published in the English language, and 13 fundamental scientific experimental studies were also excluded from consideration. As a result of this screening process, only 56 records remained eligible for retrieval. After the removal of 31 records for which either the abstracts or the full articles were inaccessible, 25 items underwent an evaluation to determine their eligibility. Furthermore, articles in which the data were either missing or inadequate for calculation purposes were excluded. Ultimately, a total of 13 studies (Ciruzzi et al., 1998; Yang et al., 2017; Tamura et al., 2018; Kim et al., 2019; Akpa et al., 2021; Cao et al., 2024; Wang et al., 2021; Shen et al., 2021; Jalali et al., 2022; Park et al., 2018; Li et al., 2015; Wu et al., 2017; Kim, Kang & Kim, 2021) satisfied the inclusion criteria. Figure 1 shows the flow diagram delineating the selection process. Moreover, the basic characteristics and NOS scores are presented in Table 1. The main types of passive smoking encompassed ETS, along with passive smoking experienced in childhood and adulthood from relatives. The identification of hypertension was mainly verified based on physical examination or according to self-reported history, including the use of antihypertensive drugs. The average NOS score of the 13 studies was 6.5. Among all of the included studies, 6 were of high quality, whereas 7 were of moderate quality.

### Impact of passive smoking on the risk of hypertension in non-smoking individuals

**Figure 2:**
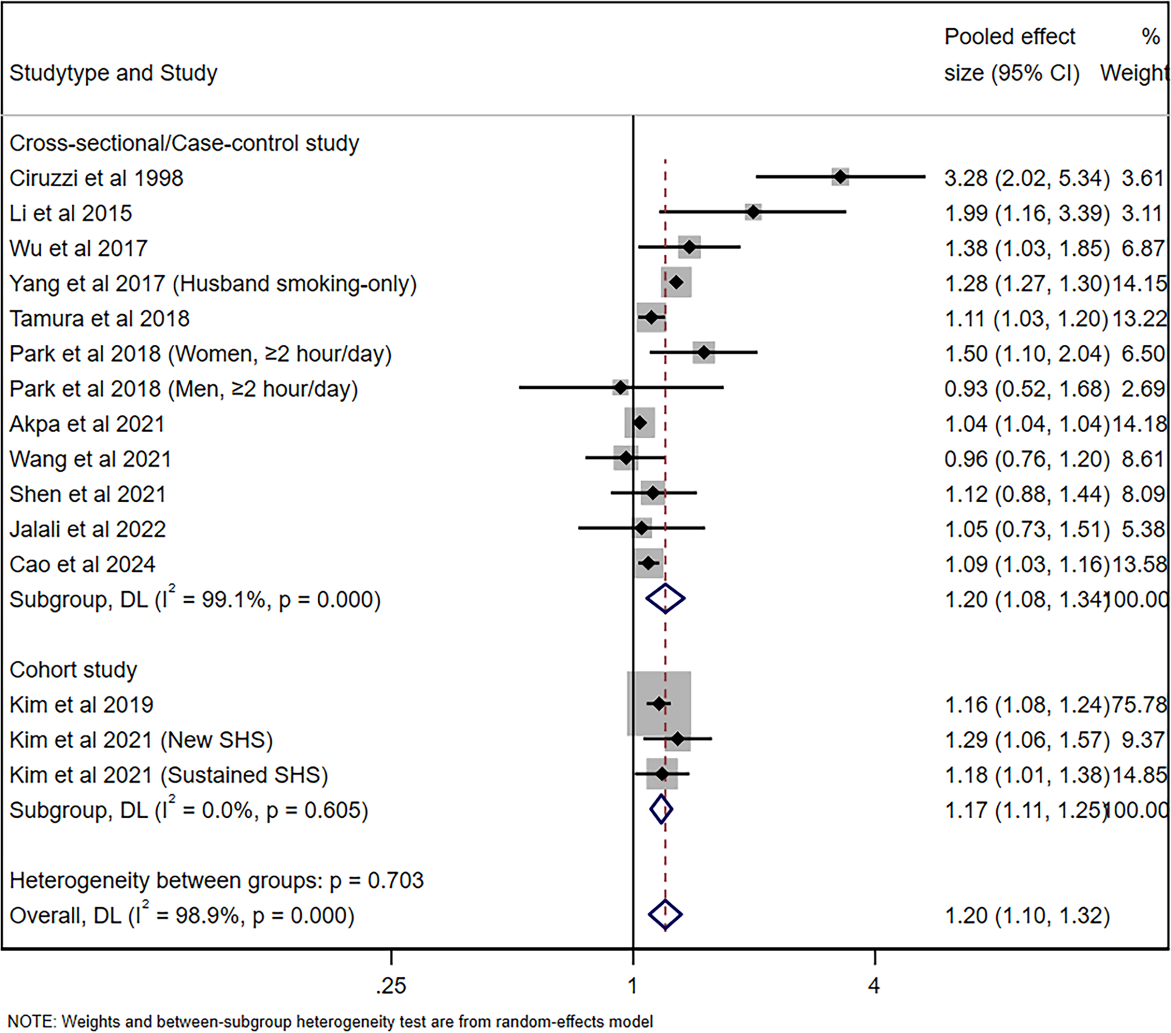
Forest plot for passive smoking and risk of hypertension (Ciruzzi et al., 1998; Li et al., 2015; Wu et al., 2017; Yang et al., 2017; Tamura et al., 2018; Park et al., 2018; Akpa et al., 2021; Wang et al., 2021; Shen et al., 2021; Jalali et al., 2022; Cao et al., 2024; Kim et al., 2019; Kim, Kang & Kim, 2021).

**Figure 3:**
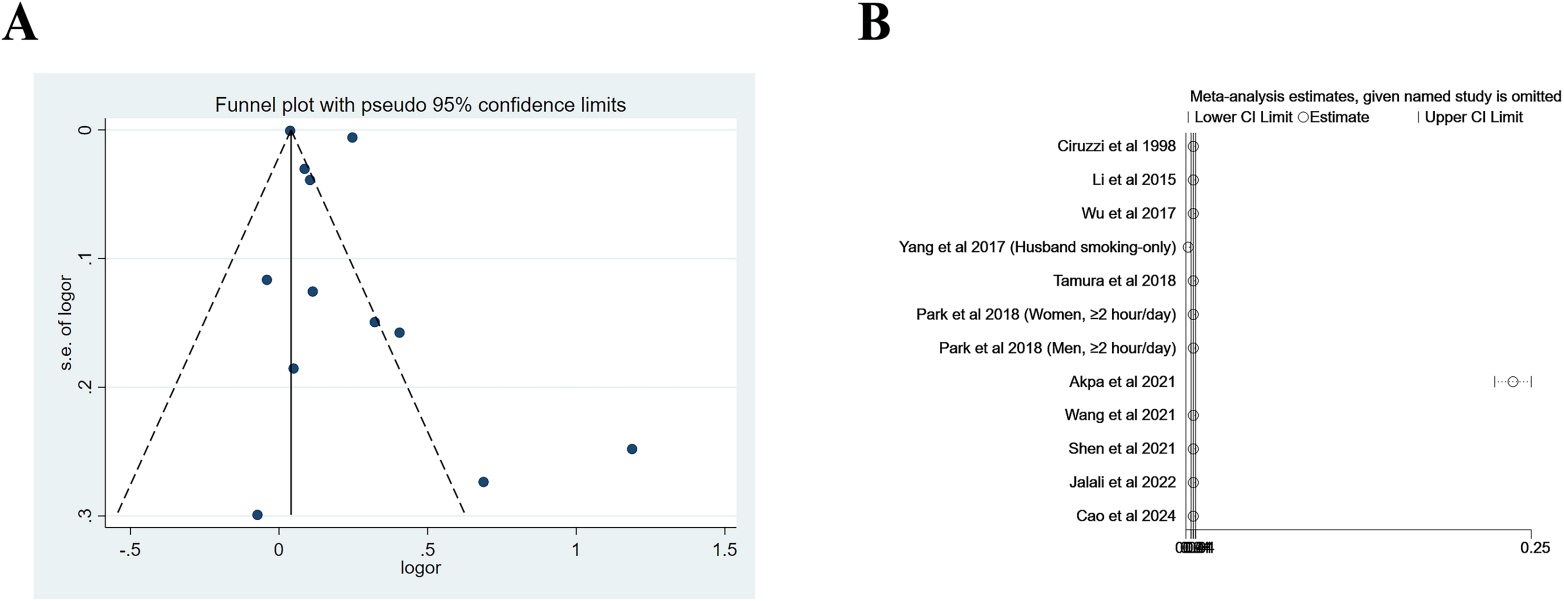
Funnel plot (A) and sensitivity analysis (B) of all included studies for cross-sectional/case-control studies.

**Figure 4:**
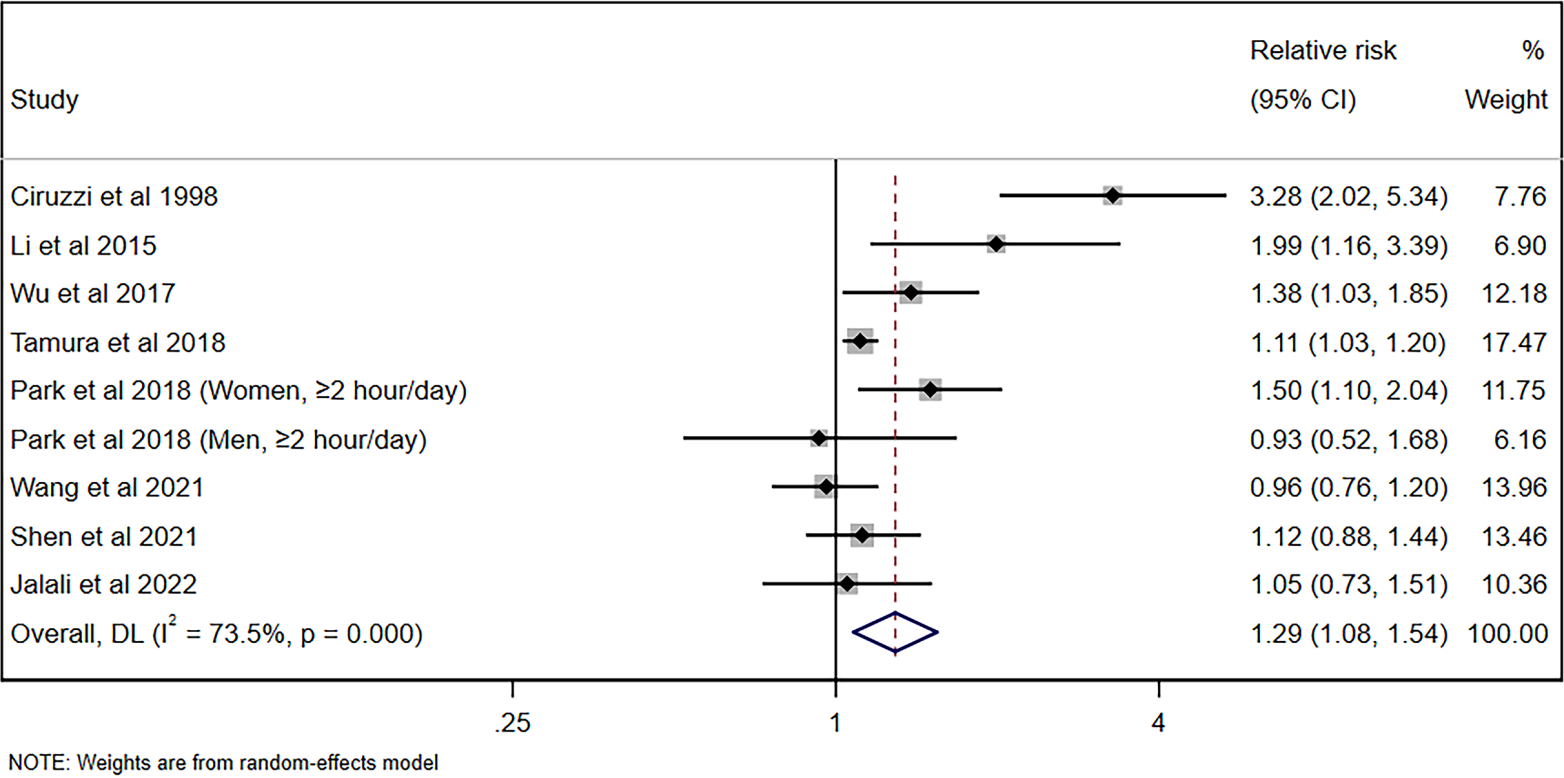
Forest plot for passive smoking and risk of hypertension with stable results for cross-sectional/case-control studies (Ciruzzi et al., 1998; Li et al., 2015; Wu et al., 2017; Tamura et al., 2018; Park et al., 2018; Wang et al., 2021; Shen et al., 2021; Jalali et al., 2022).

The pooled risk of hypertension for nonsmoking adults was 1.20 (95% CI [1.08–1.34], *p* = 0.001, *I*^*2*^= 99.1%) for cross-sectional/case-control studies and 1.17 (95% CI [1.11–1.25], *p* < 0.001, *I*^*2*^= 0%), with significant heterogeneity being observed, as shown in Fig. 2. Figure 3A shows the funnel plot for cross-sectional/case-control studies. According to the sensitivity analysis for cross-sectional/case-control studies (Fig. 3B), two studies (Yang et al., 2017; Akpa et al., 2021) were the major sources leading to the instability of the pooled analysis for cross-sectional/case-control studies. To obtain a more stable result, we excluded the abovementioned studies, and the results demonstrated that passive smoking continued to be linked to an increased risk of developing hypertension (Effect size = 1.29, 95% CI [1.08–1.54], *p* = 0.005, *I*^*2*^= 73.5%, Fig. 4).

### Subgroup analyses

**Figure 5:**
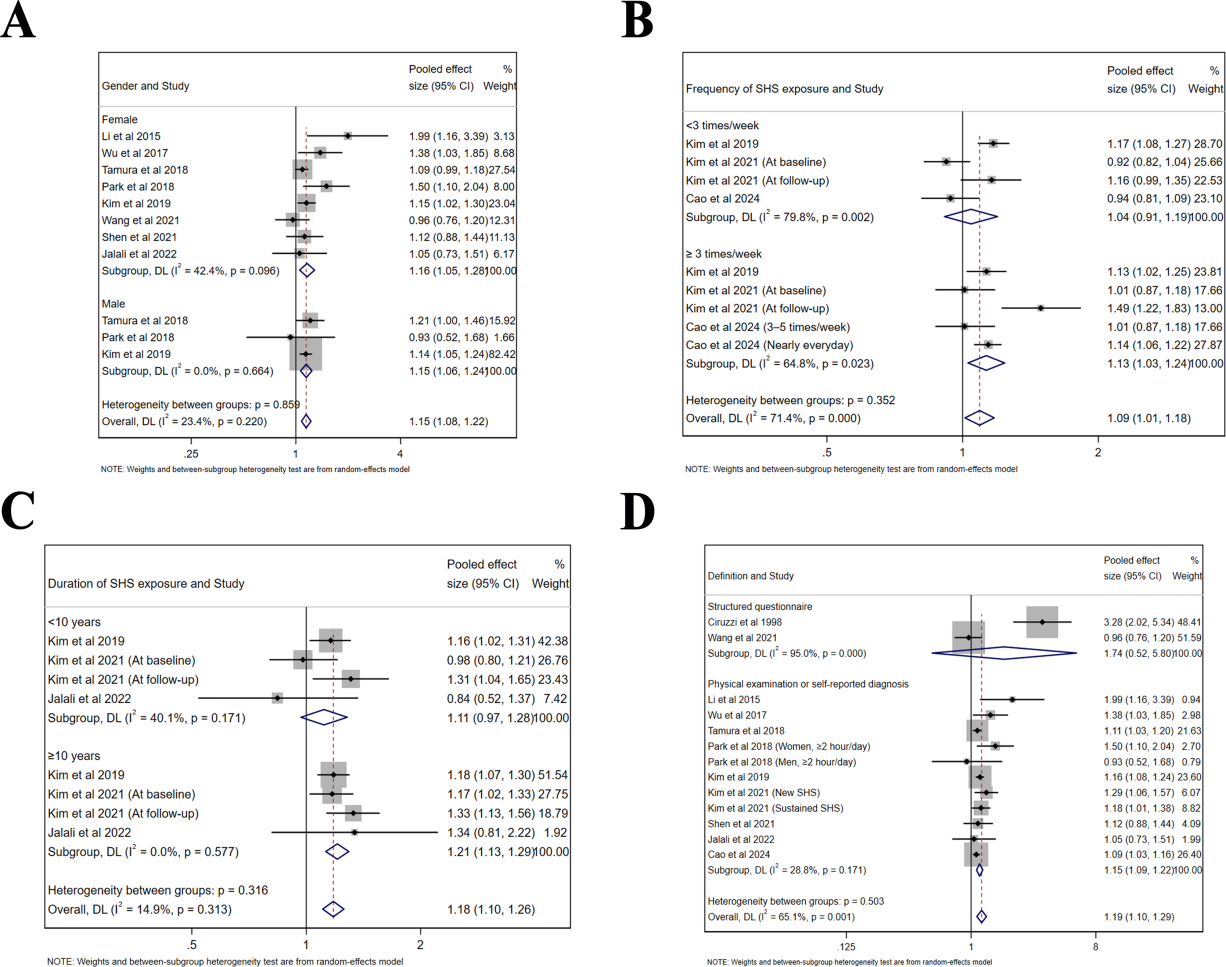
Forest plot of relative risk between passive smoking and hypertension in subgroup analysis. (A) Gender. (B) Frequency of SHS exposure. (C) Duration of SHS exposure. (D) Definition of hypertension. The risk of hypertension is increased for all genders, and in studies defined by physical examination. Exposure ≥3 times/week and ≥10 years may have an adverse influence on the risk of hypertension (Li et al., 2015; Wu et al., 2017; Tamura et al., 2018; Park et al., 2018; Kim et al., 2019; Kim, Kang & Kim, 2021; Wang et al., 2021; Shen et al., 2021; Jalali et al., 2022; Cao et al., 2024; Ciruzzi et al., 1998).

Subgroup analyses were performed based on sex, frequency and duration of SHS exposure, as well as definition of hypertension (Table 2). The same findings were confirmed in both male (Effect size = 1.15, 95% CI [1.06–1.24], *p* < 0.001, *I*^*2*^= 0.0%) and female populations (Effect size = 1.16, 95% CI [1.05–1.28], *p* = 0.003, *I*^*2*^= 42.4%, Fig. 5A). For the frequency of SHS exposure, only those who were exposed ≥3 times/week (Effect size = 1.13, 95% CI [1.03–1.24], *p* = 0.012, *I*^*2*^= 64.8%) compared to those who were exposed <3 times/week (Effect size = 1.04, 95% CI [0.91–1.19], *p* = 0.535, *I*^*2*^= 79.8%) demonstrated an increased risk of hypertension (Fig. 5B). Moreover, only exposure ≥10 years (Effect size = 1.21, 95% CI [1.13–1.29], *p* < 0.001, *I*^*2*^= 0.0%) compared to an exposure <10 years (Effect size = 1.11, 95% CI [0.97–1.28], *p* = 0.132, *I*^*2*^= 40.1%) contributed to an increased risk of hypertension (Fig. 5C). Since the definition of hypertension varied among included studies, the risk was significant for those defined by physical examination or self-reported diagnosis (Effect size = 1.15, 95% CI [1.09–1.22], *p* < 0.001, *I*^*2*^= 28.8%), but not for those by structured questionnaire with limited studies included (Effect size = 1.74, 95% CI [0.52–5.80], *p* = 0.367, *I*^*2*^= 95.0%, Fig. 5D).

**Table 2:** Subgroup analyses stratified by gender, frequency and duration of SHS exposure. The risk of hypertension is increased for all genders, and in studies defined by physical examination. Exposure ≥3 times/week and ≥10 years may have an adverse influence on the risk of hypertension.

Group	Subgroup	Pooled effect size (95% CI)	Test for overall effect (*p* value)	Heterogeneity *I*^*2*^, %
Gender	Female	1.16 [1.05–1.28]	0.003	42.4%
	Male	1.15 [1.06–1.24]	<0.001	0.0%
Frequency of SHS exposure	<3 times/week	1.04 [0.91–1.19]	0.535	79.8%
	≥3 times/week	1.13 [1.03–1.24]	0.012	64.8%
Duration of SHS exposure	<10 years	1.11 [0.97–1.28]	0.132	40.1%
	≥10 years	1.21 [1.13–1.29]	<0.001	0.0%
Definition of hypertension	Structured questionnaire	1.74 [0.52–5.80]	0.367	95.0%
	Physical examination or self-reported diagnosis	1.15 [1.09–1.22]	<0.001	28.8%

Note:

Abbreviations: SHS, secondhand smoke; CI, confidence interval.

### Quality of evidence

No high-quality evidence was identified for the outcomes assessed. The “very low” classification of the overall evidence (Table 3) was driven by fundamental concerns regarding the non-randomized designs employed, considerable heterogeneity among the analyses, and detected publication bias.

**Table 3:** Quality of evidence. The evidence is downgraded as very low.

Certainty assessment							Effect	Certainty	Importance
No. of studies	Study design	Risk of bias	Inconsistency	Indirectness	Imprecision	Other considerations	Effect size (95% CI)		
**Hypertension (cross-sectional/case-control studies)**									
10	Non-randomised studies	Serious	Not serious	Not serious	Not serious	Publication bias strongly suspected	OR 1.20 [1.08–1.34]	⨁◯◯◯ Very low	CRITICAL
**Hypertension (cohort studies)**									
3	Non-randomised studies	Serious	Not serious	Not serious	Not serious	Publication bias strongly suspected	RR 1.17 [1.11–1.25]	⨁◯◯◯ Very low	CRITICAL

Note:

CI, confidence interval; OR, odds ratio; RR, risk ratio.

## Discussion

Based on this meta-analysis, we discovered that there was a positive correlation between passive smoking and the likelihood of developing hypertension among nonsmoking adults. The risk was justifiable in both cross-sectional/case-control and cohort studies, as well as in both male and female populations. The current evidence suggests that only frequent (≥3 times/week) and long-term (≥10 years) exposure confers an increased risk of hypertension.

An earlier meta-analysis performed by Aryanpur et al. (2019) first revealed that active and passive cigarette smoking were not associated with the development of hypertension; however, they were observed to be associated with increased SBP in children and adolescents, with few studies being included. We demonstrated that passive smoking was associated with an increased risk of hypertension in nonsmoking adults. Although the exact mechanism underlying this effect is still obscure, several studies have provided evidence explaining the abovementioned association. The stiffness of large arteries and pulse wave reflection determine central BP (Laurent et al., 2006), which correspondingly affects the development of hypertensive target organ disease (Ghiadoni et al., 2009). Moreover, hazardous components from cigarette smoking can lead to an increase in the stiffness of the carotid or aortic arteries among smokers (Failla et al., 1997; Stefanadis et al., 1997). In addition, among habitual smokers, the smoking of a single cigarette results in a short-term increase in arterial wall stiffness, which can potentially cause harm to the artery and increase the risk of plaque rupture (Kool et al., 1993). In male smokers with hypertension, acute smoking of one cigarette can lead to immediate elevations in heart rate, brachial BP, and aortic pulse wave velocity (Rhee et al., 2007). In chronic smokers, acute cigarette smoking can increase central BP and affect parameters related to arterial stiffness and peripheral wave reflection (Mahmud & Feely, 2003). In accordance with the short-term effects of smoking, several studies have reported that long-term smoking is associated with arterial stiffening, both in normotensive and hypertensive individuals (Levenson et al., 1987; Liang et al., 2001).

In addition, being exposed to passive smoking over the long term has also been linked to the stiffness of the carotid artery (Mack et al., 2003). Exposure to tobacco smoke during childhood leads to a reduction in the aortic elastic properties of healthy children (Kallio et al., 2009). Moreover, a previous study demonstrated that compared with the unexposed group, adults with a body mass index (BMI) greater than 27 kg/m^2^ who were persistently exposed to SHS in domestic environments, workplaces, and various other public or private spaces exhibited more significant carotid stiffness in a dose-dependent manner (Benowitz, 2003), which significantly contributed to hypertension. Stefanadis et al. (1998) reported that passive or environmental smoking is linked to an acute decline in aortic stiffness. Additionally, when measured using the aortic pressure-diameter loop, an increase in this parameter was observed within just 4 min of passive smoking (Stefanadis et al., 1998). In addition, in healthy males, an elevation in the augmentation index (AIx), which serves as a measure of arterial stiffness and peripheral wave reflection, has also been detected (Mahmud & Feely, 2004). This increase occurred when these subjects were in an unventilated room for 1 h after being exposed to passive smoking from 15 cigarettes (Mahmud & Feely, 2004). However, the detailed mechanisms underlying these effects still require further investigation.

The association was further verified *via* subgroup analysis. With respect to study type and sex, the results from all types of studies, as well as those from both male and female populations, were in agreement. This consistency suggests that neither the study type nor sex has an effect on the overall outcomes. These findings further reinforce the soundness and reliability of the conclusions. Regarding the frequency and duration of SHS exposure, the current evidence suggests that only frequent and sustained exposure could be a risk factor for hypertension, with little heterogeneity being observed, thereby indicating that the association may be dose- and time-dependent. This conclusion does not necessarily imply that minimal exposure to SHS lacks an adverse effect on hypertension. However, due to the limited number of studies that were included in the abovementioned subgroup analysis, more well-designed and population-based studies are needed to verify these associations.

Our study has several limitations. Despite adjusted estimates being utilized across the included studies, unmeasured confounding factors and biases persisted, which included genetic variations, lifestyle-associated factors, and the exclusion of non-English language articles. In addition, based on the GRADE assessment, the certainty of the evidence was extremely low. This was partially attributed to the nonrandomized study design and the potential presence of publication bias. In certain subgroup analyses, the quantity of included studies remained restricted. This was particularly evident in aspects such as the frequency and duration of SHS exposure. Consequently, it is necessary to conduct more population-based studies to validate these correlations.

## Conclusions

In summary, we discovered a notable connection between passive smoking and the risk of hypertension in cross-sectional/case-control and cohort studies, as well as in male and female populations. Current evidence indicates that only frequent (≥3 times/week) and long-term (≥10 years) exposure to passive smoking has the potential to increase the risk of hypertension. Our meta-analysis offers additional considerations that should be addressed when formulating prevention and surveillance strategies for hypertension.

## Supplemental Information

10.7717/peerj.20639/supp-1 Supplemental Information 1 PRISMA checklist.

10.7717/peerj.20639/supp-2 Supplemental Information 2 Data extraction and quality assessment of studies by Newcastle-Ottawa Scale (NOS).

10.7717/peerj.20639/supp-3 Supplemental Information 3 Original data.

## Additional Information and Declarations

### Data Availability

The following information was supplied regarding data availability:

Raw data was not generated in this systematic review/meta-analysis.
