# Supplementary material for: Passive smoking and the risk of hypertension in nonsmoking adults: a systematic review and meta-analysis
Source: PeerJ. 2026 Jan 14;14:e20639. doi: 10.7717/peerj.20639 (PMC12811966; doi:10.7717/peerj.20639)
Supplement: Supplemental Information 2 [file peerj-14-20639-s002.docx]

**Supplementary Table 1 Data extraction and quality assessment of studies by Newcastle-Ottawa Scale (NOS)**

| **Study** | **OR/RR** | **95%CI**  **Lower Limit** | **95%CI**  **Upper Limit** | **Selection** | **Comparability** | **Outcome** | **Total NOS** |
| --- | --- | --- | --- | --- | --- | --- | --- |
| Ciruzzi et al 1998 | 3.28 | 2.02 | 5.34 | ★★ | ★★ | ★★ | 6 |
| Li et al 2015 | 1.99 | 1.16 | 3.39 | ★★ | ★★ | ★★ | 6 |
| Wu et al 2017 | 1.38 | 1.03 | 1.85 | ★★ | ★★ | ★★ | 6 |
| Yang et al 2017 | 1.28 | 1.27 | 1.30 | ★★ | ★★ | ★★ | 6 |
| Tamura et al 2018 | 1.11 | 1.03 | 1.20 | ★★★ | ★★ | ★★ | 7 |
| Park et al 2018 (Women) | 1.50 | 1.10 | 2.04 | ★★★ | ★★ | ★★ | 7 |
| Park et al 2018 (Men) | 0.93 | 0.52 | 1.68 |  |  |  |  |
| Kim et al 2019 | 1.16 | 1.08 | 1.24 | ★★★ | ★★ | ★★ | 7 |
| Akpa et al 2021 | 1.038 | 1.037 | 1.040 | ★★★ | — | ★★ | 5 |
| Wang et al 2021 | 0.96 | 0.76 | 1.20 | ★★ | ★★ | ★★ | 6 |
| Kim et al 2021  (New SHS) | 1.29 | 1.06 | 1.57 | ★★★★ | ★★ | ★★ | 8 |
| Kim et al 2021  (Sustained SHS) | 1.18 | 1.01 | 1.38 |  |  |  |  |
| Shen et al 2021 | 1.12 | 0.88 | 1.44 | ★★ | ★★ | ★★ | 6 |
| Jalali et al 2022 | 1.05 | 0.73 | 1.51 | ★★ | ★★ | ★★ | 6 |
| Cao et al 2024 | 1.09 | 1.03 | 1.16 | ★★★ | ★★ | ★★ | 7 |

★ represents one point in Newcastle-Ottawa Scale scoring system.

OR, odds ratio; RR, relative risk; CI, confidential interval; SHS, secondhand smoking.
